# Chemotherapy regimens for advanced pancreatic cancer: a systematic review and network meta-analysis

**DOI:** 10.1186/1471-2407-14-471

**Published:** 2014-06-27

**Authors:** Gillian K Gresham, George A Wells, Sharlene Gill, Christopher Cameron, Derek J Jonker

**Affiliations:** 1 Department of Epidemiology and Community Medicine, University of Ottawa, Ottawa, Ontario, Canada; 2 British Columbia Cancer Agency, Vancouver, British Columbia, Canada; 3 The Ottawa Hospital Cancer Centre, Ottawa, Ontario, Canada

**Keywords:** Advanced pancreatic cancer, Chemotherapy, Gemcitabine, Combination therapy, Randomized clinical trials, Systematic review, Network meta-analysis

## Abstract

**Background:**

Advanced pancreatic cancer confers poor prognosis and treatment advancement has been slow. Recent randomized clinical trials (RCTs) have demonstrated survival benefits for combination therapy compared to gemcitabine alone. However, the comparative benefits and harms of available combination chemotherapy treatments are not clear. We therefore conducted a systematic review and Bayesian network meta-analysis to assess the comparative safety and efficacy of chemotherapy regimens for the treatment of advanced pancreatic cancer.

**Methods:**

MEDLINE, PubMed, EMBASE, Cochrane Central Registry of Clinical trials and abstracts from major scientific meetings were searched for RCTs published from 2002 to 2013. Key outcomes were overall survival (OS), progression free survival (PFS), and safety including grade 3–4 febrile neutropenia, neutropenia, vomiting, diarrhea, fatigue and sensory neuropathy. Bayesian network meta-analyses were conducted to calculate survival and safety outcomes using gemcitabine (GEM) as the reference comparator. Effect estimates and 95% credible intervals were calculated for each comparison. Mean ranks and the probability of being best were obtained for each treatment analyzed in the network meta-analysis.

**Results:**

The search identified 23 studies involving 19 different treatment regimens and 9,989 patients. FOLFIRINOX, GEM/cisplatin/epirubicin/5FU (PEFG), GEM/NAB-paclitaxel (NAB-P), GEM/erlotinib+/-bevacizumab, GEM/capecitabine, and GEM/oxaliplatin were associated with statistically significant improvements in OS and PFS relative to gemcitabine alone and several other treatments. They were amongst the top ranked for survival outcomes amongst other treatments included. No significant differences were found for other combination chemotherapy treatments. Effect estimates from indirect comparisons matched closely to estimates derived from pairwise comparisons. Overall, combination therapies had greater risk for evaluated grade 3–4 toxicities over gemcitabine alone.

**Conclusions:**

In the absence of head-to-head comparisons, we performed a mixed-treatment analysis to achieve high-quality information on the effectiveness and safety of each treatment. This study suggests that some combination therapies may offer greater benefits in the treatment of advanced pancreatic cancer than others. To more fully elucidate the comparative benefits and harms of different combination chemotherapy regimens, rigorously conducted comparative studies, or network meta-analysis of patient-level data are required.

## Background

Pancreatic adenocarcinoma is the fourth leading cause of cancer death in North America [1]. In the United States, there will be 45 220 new cases in 2013, with 38 460 deaths due to pancreatic cancer [2]. Prognosis is poor with a five-year overall survival (OS) rate of 5% for all cases. Due to the insidious nature of the disease, 80-85% of patients will be diagnosed with advanced disease at presentation, where the five-year OS rate drops to only 2% [1].

While supportive care measures such as opioids, radiotherapy and nerve blocks are critical for optimal symptom management in patients with advanced disease, systemic chemotherapy has had the greatest impact on survival. Since the approval of gemcitabine (GEM) as the standard first-line therapy in 1997, several new systemic regimens have been investigated to treat this population [3]. However, only modest improvements in survival outcomes have been observed [4]. Agents which have been investigated in combination with GEM included oxaliplatin, capecitabine, cisplatin or 5-fluorouracil as well as GEM-based biologic therapies, erlotinib and more recently the combination of erlotinib and bevacizumab [5-28]. In 2005, a four-drug regimen, including GEM, cisplatin, epirubicin, 5-fluorouacil (PEFG) demonstrated improved overall survival (OS) and progression free survival (PFS) over GEM alone [19]. In 2011, a four drug regimen, which included folinic acid, 5-fluorouacil, irinotecan and oxaliplatin (FOLFIRINOX), was shown to have significantly superior survival outcomes compared to GEM alone [29]. This resulted in the adoption of FOLFIRINOX as the preferred option for patients with good performance status (ECOG 0-1/KPS > 70). However, there is controversy as to whether the survival benefits of four drug combination regimens outweigh the associated toxicities. More recently, a trial comparing GEM/NAB-P versus GEM alone demonstrated a statistically significant survival benefit for this new doublet, introducing another option for the management of advanced pancreatic cancer [28]. With the introduction of these therapeutic options, and the lack of randomized trials that directly compare all available treatments, it was of interest to indirectly compare the relative efficacy and safety of these treatments using a network meta-analysis.

The objective was to perform a comprehensive systematic review of all phase III randomized clinical trials published over the last decade comparing GEM to combination therapies for patients with advanced pancreatic cancer and compare the relative efficacy and safety of these treatments using a Bayesian network meta-analysis. The network meta-analysis incorporates both direct and indirect comparisons, using GEM as the reference comparator, in order to compute the hazard ratios (HR) for OS, and safety outcomes between all treatments on a relative scale. This analysis also provides information about the rankings of various treatments in terms of survival outcomes and safety.

## Methods

### Identification of randomized studies

Randomized studies in any language were searched using Medline, EMBASE, PubMed and the Cochrane Central Registry of Controlled Trials over the past decade. The search strategy included the key words “advanced OR metastatic AND pancreatic AND (‘cancer’/exp OR cancer) OR ‘adenocarcinoma’/exp OR adenocarcinoma OR pancrea* OR malign* AND (‘neoplasm’/exp OR neoplasm) AND ‘randomized controlled trial’/de AND ‘pancreas cancer’/de and was further filtered in an advanced search for randomized clinical trials from 2002–2013. Limits included phase III randomized clinical trials. Abstract presentations of the American Society of Clinical Oncology (ASCO) and the European Society of Clinical Oncology were searched in order to identify any phase III trials that had not been published. The reference lists of existing systematic reviews and clinicaltrials.gov were cross-referenced against our search results in order to identify any additional RCTs. Two authors (GG and SG) independently screened the abstracts and selected eligible trials. Any discrepancies were discussed with a third reviewer (DJ). Selected studies were then assessed for bias and overall study quality using the SIGN 50 assessment scale [30].

### Eligibility criteria

Randomized clinical trials with at least two arms comparing different chemotherapy regimens in patients with advanced pancreatic cancer from January 1st 2002-January 31^st^ 2013 were considered. Clinical trials comparing chemotherapy either in the form of monotherapy or combination therapy were included if they were either directly or indirectly connected to the reference comparator, GEM, and if they enrolled at least 50 patients per arm based on the recommendations from the literature [31]. The trial population included patients who were eligible for first-line therapy and who were diagnosed with metastatic disease. Trials including over 50% of patients with locally advanced non-metastatic disease in their treatment arms were excluded from this study because treatment approaches, response to therapy and outcomes for locally advanced disease may differ from metastatic disease. Trials involving radiation therapy were also excluded to avoid clinical heterogeneity. Phase II trials were excluded from this study as primary outcomes differ for the majority of these studies, and potential heterogeneity and bias is further introduced due to the lack of blinding and smaller sample sizes of these studies. Finally, trials including histology other than adenocarcinoma (e.g. neuroendocrine tumours) were excluded. Interventions of interest included any single-agent or combination chemotherapy where the comparators were head-to-head. Outcomes of interest were OS, PFS and safety.

### Data extraction

Trial data was collected from the original publication including the authors’ names, the journal, year of publication, country of origin, number of participating centers, inclusion and exclusion criteria, stratification, major and minor endpoints, number of arms, sample size per arm, regimens used, doses and line of treatment using a piloted data extraction form. Patient characteristics were documented including the ratio of males to females, proportion of stage IV disease and the proportion of good ECOG or KPS performance status. Survival outcomes were assessed from published HR and 95% credible intervals. Where multiple publications existed for a single randomized clinical trial, only results from the most recent adjudicated publication were used in the analysis. The Scottish Intercollegiates Guidelines Network (SIGN) 50 assessment scale was used in order to determine the overall methodological quality of the studies [30]. This was based on answers about sources of funding, internal validity and risk of bias.

### Outcome measures

The primary outcome was OS, calculated as the date of randomization until the date of death. Secondary outcomes included PFS and safety. PFS was calculated as the date of randomization until the date of documentation of disease progression or death. Differences in the time-dependent survival outcomes were computed as log HRs within the network meta-analysis. The ORR was calculated from the proportion of complete and partial responses as defined in the ERTCC v 3.0 and divided by the total number of patients per arm. Grade 3 (serious) or 4 (life-threatening) adverse events of interest were specified *a priori* and included febrile neutropenia, neutropenia, fatigue, vomiting, diarrhea and sensory neuropathy as defined in the Common Terminology Criteria in Adverse Events (CTCAE) v 3.0 [32]. ORR and safety were compared using odds ratios.

### Statistical analysis

Descriptive statistics were generated for trial and study population characteristics across all eligible trials using SAS (9.2; Cary, NC). Median values were obtained for each characteristic per arm when applicable, and overall trial proportions were calculated from data provided in the trial’s study characteristics.

Pairwise comparisons were generated by synthesizing studies that compared the same interventions into a random effects model. Random effects models were used for the pairwise comparison with the exception of the use of fixed effects models for comparisons in which only a single study was included for that particular treatment comparison. The pooled hazard ratios and 95% confidence intervals were then reported for the outcomes of interest. All statistical analyses of the meta-analysis were conducted using RevMan [5.2, Cochrane Collaboration, Copenhagen] [33].

A Bayesian network meta-analysis was performed in order to simultaneously compare all treatments in the network. The network meta-analysis can be thought of as an extension of the traditional meta-analysis, as it incorporates both direct and indirect information through a common comparator in order to obtain estimates of the relative treatment effects on the multiple treatment comparisons [34-37]. For instance, by obtaining information from a trial comparing drug A to B, and B to C, an indirect estimate of the benefit of A over C can be achieved [36]. A normal likelihood model incorporating log hazard ratios of treatment differences was used for the analyses. Bayesian methods combine a prior probability distribution with a distribution of the pooled effect based on the observed data in order to obtain a posterior probability distribution of the pooled effect [35,37,38]. The resulting posterior distribution allows for its interpretation in terms of probabilities where the probability of a treatment resulting in a smaller or larger increase of survival can be determined. Furthermore, the posterior results are not influenced by the prior distribution because non-informative prior distributions are being used prior to seeing the data, and thus, the posterior distribution is driven completely by the data [35]. The Bayesian framework for network meta-analyses also allows for the probabilistic interpretation of uncertainty and ranking of interventions [39]. Therefore, it makes it possible to identify the most effective treatment and to rank treatments in order of effectiveness and tolerability.

Gemcitabine, an established standard therapy for advanced pancreatic cancer, was selected as the reference comparator in the Bayesian network meta-analysis because it has consistently been used as the comparator in the majority of randomized clinical trials available for advanced pancreatic cancer. Following assessment of heterogeneity across trials in terms of patient characteristics, trial methodologies, and treatment protocols, point estimates and 95% credible intervals were generated. Credible intervals represent the extent of uncertainty around the point estimate and thus can be interpreted as the probabilistic statement about the parameter [35]. Absolute prolongation of survival with various regimens for a patient was calculated based on the median survival of GEM, the standard therapy and reference comparator for the network meta-analysis. It was calculated as [(GEM median OS/Hazard Ratio)-GEM median OS].

The probability of a comparator being optimal was estimated for each outcome and the mean rank was calculated, by counting the proportion of iterations of the Markov chain in which each drug had the highest hazard ratio. Vague or flat priors, such as N (0, 100^2^) were assigned for basic parameters throughout [40]. Outcomes were compared from the fixed and random effects models and reported estimates from the model with a better fit, which was based on the deviance information criterion and comparing the residual deviance with the number of unconstrained data points. To ensure convergence was reached, trace plots and the Brooks-Gelman-Rubin statistic were assessed. Three chains were fit in WinBUGS for each analysis, with at least 40,000 iterations, and a burn-in of at least 40,000 iterations [40]. All Bayesian network meta-analyses were conducted in WinBUGS 1.4 (MRC Biostatistics Unit, Cambridge, UK).

Clinical heterogeneity was first assessed through clinical judgment with input from experts in the field. Statistical heterogeneity was then assessed by visually inspecting forest plots from pairwise analysis to determine whether there was overlap in the confidence intervals, as this would suggest heterogeneity. A formal assessment of heterogeneity was then accomplished by referring to the I^2^ statistic. Following standard guidelines, I^2^ values greater than 50% are considered high heterogeneity levels, between 25-50%, moderate and less than 25%, considered low heterogeneity levels. In instances where heterogeneity was suspected, sensitivity analysis was employed.

Sensitivity analyses were conducted to adjust for important covariates based the suspicion of heterogeneity from either the clinical or statistical assessments of heterogeneity, as described in the previous sections.

Covariates that were selected to be analyzed in the Bayesian network meta-analysis, *a priori*, included patient performance status, years of publication, trial sample size and the proportion of stage IV disease versus locally advanced. The sensitivity analysis for patient performance status excluded trials conducted with a proportion of patients with greater than 85% ECOG PS 0-1/KPS of 90–100, based on clinical recommendations. The sensitivity analysis for trial size utilized a threshold value of 100 patients/arm, based on recommendations from Juni et al. [37]. The sensitivity analysis for stage mix (locally advanced versus metastatic) excluded trials with 80% or fewer of patients with stage 4 disease. The analysis for year of publication excluded any trials conducted prior to 2007.

## Results

### Description of eligible trials

**Figure 1:**
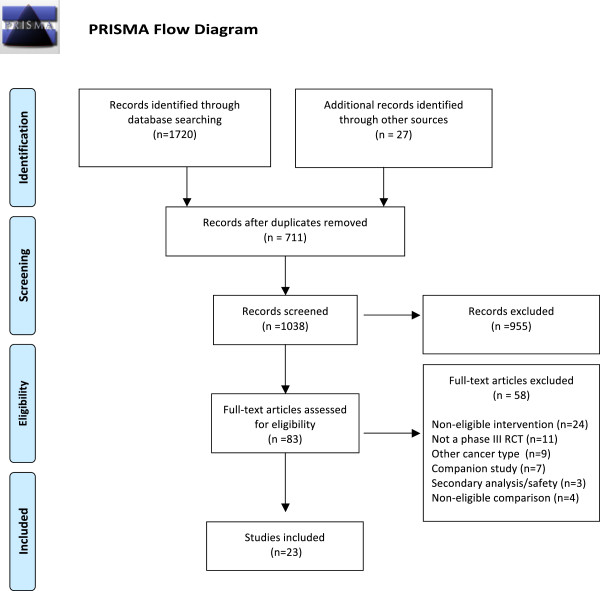
Flow chart of randomized controlled clinical trials evaluating treatments for advanced pancreatic cancer through selection process.

The initial search of the population resulted in a total of 1747 studies. After removal of duplicates and title/abstract screening, 83 trials were eligible for full-text screening resulting in 23 trials that were included in the study (Figure 1). A search of major scientific meetings yielded two additional abstracts that were included in the systematic review and network meta-analysis.

**Figure 2:**
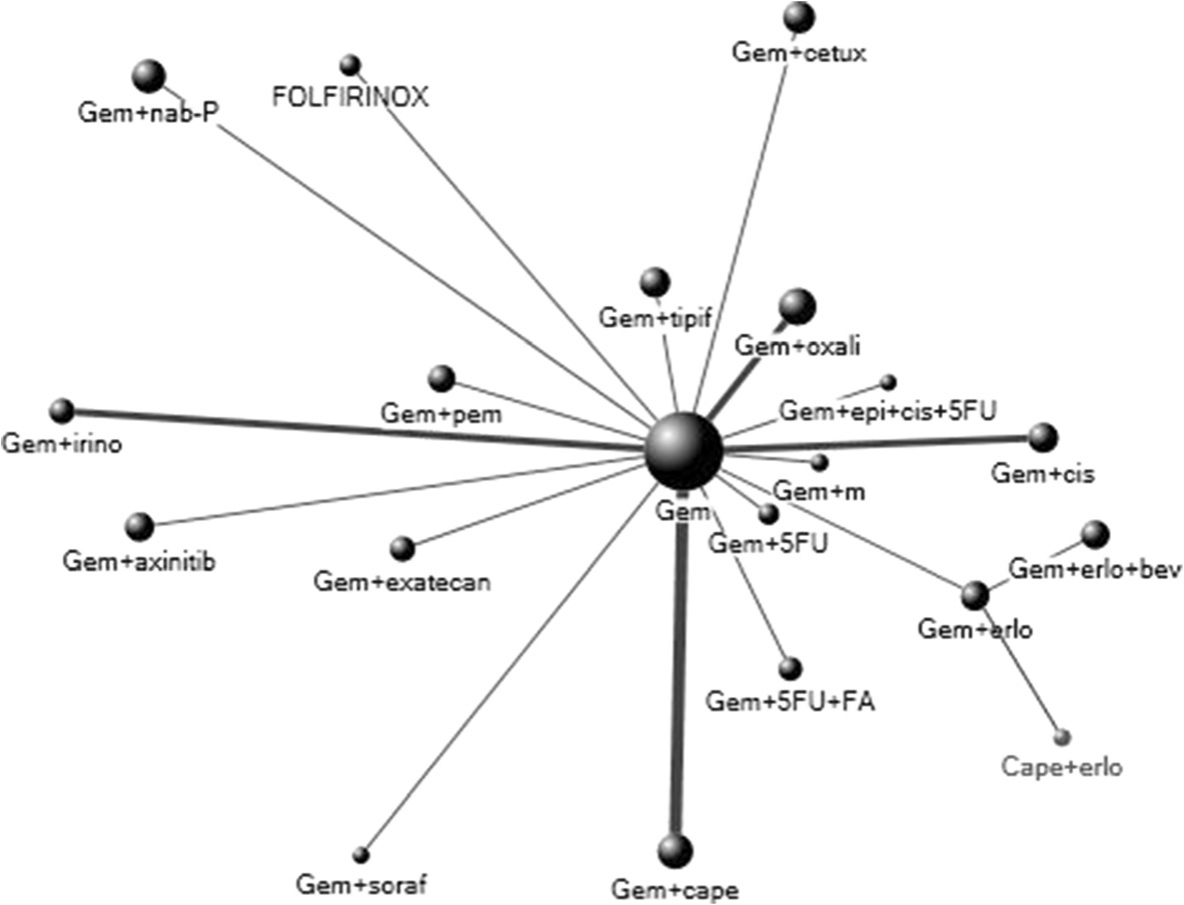
**Network of eligible trials where center node represents the reference comparator: Gemcitabine.***Circle size is proportionate to the number of patients, thickness of line represents number of trials per comparison and distance of circle to reference comparator (gemcitabine) is proportionate to year of publication where 1 cm = 2 years. Gem = gemcitabine. 5FU = 5-fluorouacil. FA = Folinic Acid. Erlo = erlotinib. Epi = epirubicin. Bev = bevacizumab. Cape = capecitabine. Soraf = sorafinib. M = marismastat. Cis = cisplatin. Irino = irinotecan. Oxali = oxaliplatin. Tipif = tipifarnib. Cetux = cetuximab. NAB-P = NAB-Paclitaxel.*

Characteristics of the included trials are outlined in Table 1. A total of 9989 randomized patients were included in the analysis. The majority of the trials had two arms and compared GEM to an experimental treatment. Individual trial arms were evenly distributed between age, gender and performance status. Using the SIGN 50 scale, 5/23 studies (21.7%) were reported as high quality and the remaining 18 studies (78.3%) as acceptable quality studies. For the primary outcomes of interest, 19 unique comparisons were available for 23 different trials. The resulting network geometry is depicted in Figure 2.

**Table 1:** Characteristics of eligible randomized clinical trials included network meta-analysis

**Study: Author (year)**	**Study design: number of patients**	**Regimens: arm 1**	**Regimens: arm 2**	**Outcomes**	**Publication type**	**Quality (Sign50)**
Bramhall (2002)	RCT-double blinded	Gemcitabine (1000 mg/m^2^)	Gemcitabine + Marismastat	OS	Full-text	++
	N1 = 119			PFS		
	N2 = 120			ORR		
Berlin (2002)	RCT-single blinded	Gemcitabine (1000 mg/m^2^)	Gemcitabine + 5FU	OS	Full-text	+
	N1 = 162			PFS		
	N2 = 160			ORR		
VanCustem (2004)	RCT- double blinded	Gemcitabine (1000 mg/m^2^)	Gemcitabine + Tipifarnib	OS	Full-text	++
	N1 = 344			PFS		
	N2 = 344			ORR		
Rocha Lima (2004)	RCT- single blinded	Gemcitabine (1000 mg/m^2^)	Gemcitabine + Irinotecan	OS	Full-text	+
	N1 = 180			ORR		
	N2 = 180					
Louvet (2005)	RCT- single blinded	Gemcitabine (1000 mg/m^2^)	Gemcitabine + Exatecan	OS	Full-text	+
	N1 = 156			PFS		
	N2 = 157			ORR		
Reni (2005)	RCT- single blinded	Gemcitabine (1000 mg/m^2^)	Gemcitabine + Oxaliplatin	OS	Full-text	+
	N1 = 47			PFS		
	N2 = 52			ORR		
Riess (2005)	RCT- single blinded	Gemcitabin (1000 mg/m^2^)	Gemcitabine + epirubicin + cisplatin + 5FU	OS	Abstract	+
	N1 = 238			PFS		
	N2 = 235			ORR		
Herrmann (2007)	RCT- single blinded	Gemcitabine (1000 mg/m^2^)	Gemcitabine + 5FU + folinic acid	OS	Full-text	+
	N1 = 159			PFS		
	N2 = 160			ORR		
Oettle (2005)	RCT- single blinded	Gemcitabine (1000 mg/m^2^)	Gem + Capecitabine	OS	Full-text	+
	N1 = 282			PFS		
	N2 = 283			ORR		
Abou-Alfa (2006)	RCT- single blinded	Gemcitabine (1000 mg/m^2^)	Gem + Pemetrexed	OS	Full-text	+
	N1 = 175			ORR		
	N2 = 1175					
Heinemann (2006)	RCT- single blinded	Gemcitabine (1000 mg/m^2^)	Gemcitabine + Cisplatin	OS	Full-text	+
	N1 = 97			PFS		
	N2 = 98					
				ORR		
Stathopoulos (2006)	RCT- single blinded	Gemcitabine (1000 mg/m^2^)	Gemcitabine + Irinotecan	OS	Full-text	+
	N1 = 70			ORR		
	N2 = 60					
Poplin (2006)	RCT- single blinded	Gemcitabine (1000 mg/m^2^)	Gemcitabine + Oxaliplatin	OS	Full-text	+
	N1 = 275			PFS		
	N2 = 272			ORR		
Moore (2007)	20 RCT- double blinded	Gemcitabine (1000 mg/m^2^)	Gemcitabine + Erlotinib	OS	Full-text	++
				PFS		
	N1 = 285					
	N2 = 284			ORR		
Cunningham (2009)	RCT- single blinded	Gemcitabine (1000 mg/m^2^)	Gem + Capecitabine	OS	Full-text	+
	N1 = 266			PFS		
	N2 = 267			ORR		
VanCustem (2009)	RCT- Double blinded	Gemcitabine + Erlotinib	Gem + Erlotinib + Bevacizumab	OS	Full-text	++
	N1 = 301			PFS		
	N2 = 306			ORR		
Philip (2010)	RCT- single blinded	Gemcitabine (1000 mg/m^2^)	Gemcitabine + Cetuximab	OS	Full-text	++
	N1 = 371			PFS		
	N2 = 372			ORR		
Colucci (2010)	RCT- single blinded	Gemcitabine (1000 mg/m^2^)	Gem + Cisplatin	OS	Full-text	+
	N1 = 199			PFS		
	N2 = 201			ORR		
Kindler (2011)	RCT-Double blinded	Gemcitabine (1000 mg/m^2^)	Gem + Axinitib	OS	Full-text	++
	N1 = 315			PFS		
	N2 = 180			ORR		
Conroy (2011)	RCT- single blinded	Gemcitabine (1000 mg/m^2^)	FOLFIRINOX	OS	Full-text	+
	N1 = 171			PFS		
	N2 = 171			ORR		
Goncalves (2012)	RCT- double blinded	Gemcitabine (1000 mg/m^2^)	Gem + Sorafenib	OS	Full-text	++
	N1 = 52			PFS		
	N2 = 52			ORR		
Heinemann (2012)	RCT-single blinded	Gemcitabine + Erlotinib	Capecitabine + Erlotinib	OS	Full-text	+
	N1 = 143			PFS		
	N2 = 131			ORR		
Von Hoff (2013)	RCT- single blinded	Gemcitabine (1000 mg/m^2^)	Gem + NAB-P	OS	Abstract	+
	N1 = 430			PFS		
	N2 = 431			ORR		

### Results from pairwise comparisons

Pairwise comparisons were accomplished for the 19 different comparisons. The weighted hazard ratios for the primary outcome, OS, were calculated for each comparison. Statistical heterogeneity was assessed using the I^2^ statistic, which was assessable in two of the comparisons, as the majority of treatments had only been tested in phase III trials once. The I^1^ values were 0% for the comparison of GEM/capecitabine *versus* GEM alone and GEM/cisplatin *versus* GEM alone. In pairwise comparisons, the combination of GEM/capecitabine, GEM/oxaliplatin, PEFG, GEM plus NAB-paclitaxel (NAB-P), GEM/erlotinib+/-bevacizumab and FOLFIRINOX were associated with statistically significant hazard ratios for OS over GEM alone (Additional file 1: Figure S1).

### Results from the network meta-analysis of the primary outcome

The effect estimates from both the fixed and random-effects models were comparable and matched closely to the estimates derived from the pairwise comparisons in both direction and magnitude.

**Figure 3:**
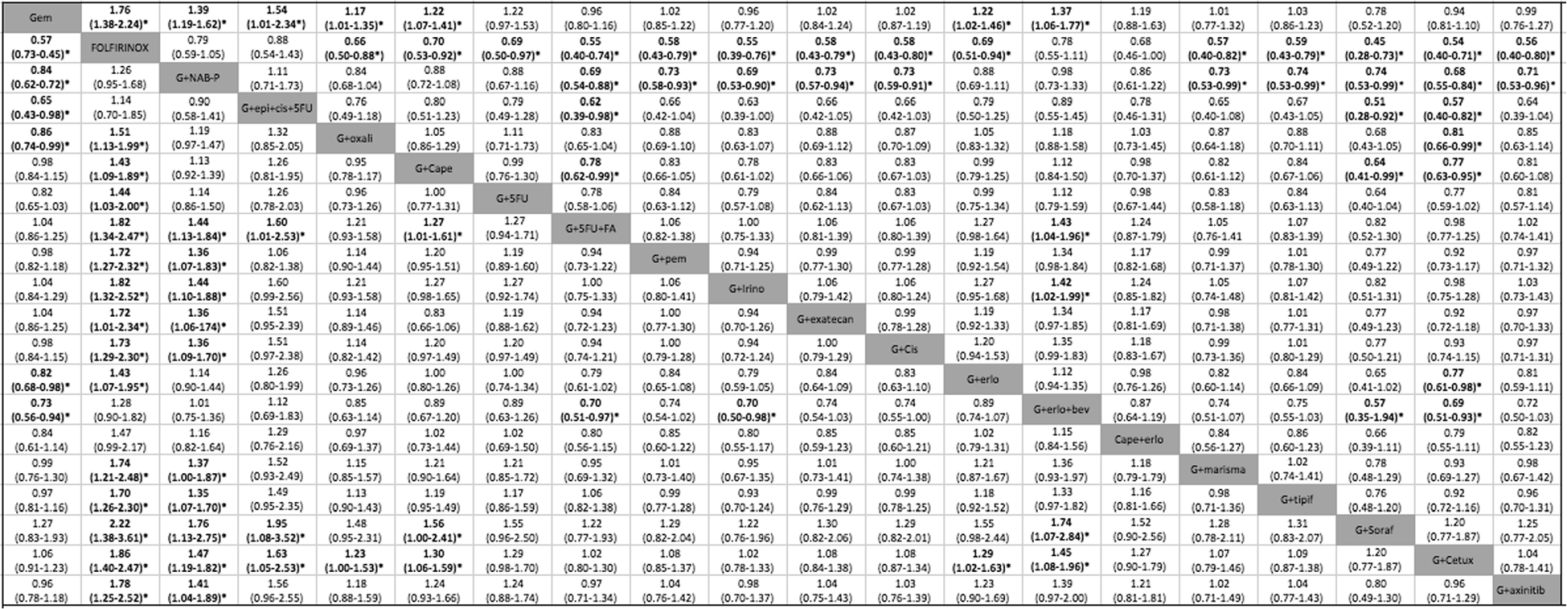
**Indirect comparisons for overall survival: HRs and 95% CIs for various treatment comparisons.** Horizontal: Experimental vs. Control; Vertical: Control vs. Experimental. HR < 1 indicates OS or PFS benefit. *Hazard ratios are statistically significant. Gem = gemcitabine; 5FU = 5-flurouacil; PEFG = gemcitabine + epirubicin + 5FU + cisplatin; NAB-P = NAB-P; bev = bevacizumab; erlo = erlotinib; cape = capecitabine; oxali = oxaliplatin; tipif = tipifarnib; pem = pemetrexed; cis = cisplatin; irino = irinotecan; FA = folinic acid; cetux = cetuximab; soraf = sorafenib.

Figure 3 illustrates the hazard ratios for OS and 95% credible intervals obtained from the indirect comparisons of the included regimens. Following Figure 3 from left to right, FOLFIRINOX; PEFG; GEM/NAB-P; GEM/erlotinib/bevacizumab; GEM/erlotinib; GEM/capecitabine and GEM/oxaliplatin were found to have significantly improved survival estimates in comparison to GEM alone. FOLFIRINOX was associated with statistically significant hazard ratios for OS relative to fifteen different treatments including GEM alone and the combinations of GEM with oxaliplatin; capecitabine; cisplatin; 5-fluorouacil+/-folinic acid; pemetrexed; irinotecan; exatecan; axinitib; tipifarnib; marimastat and sorafenib (Additional file 2: Figure S2, Additional file 3: Figure S3). FOLFINOX had a calculated OS gain of 4.2 months (95% Cl 2.2-6.9) over GEM alone and a median survival advantage of 4 months (range 0.8-6.9 months) over the other treatments included in the analysis (Table 2). FOLFIRINOX had a 64.9% probability of being best for OS (Additional file 2: Figure S2 and Additional file 4: Figure S4). Using the mean rank scale, FOLFIRINOX was ranked first with a mean rank of 1.5 out of 20 treatments (Additional file 3: Figure S3). FOLFIRINOX was not associated with statistically significant hazard ratios for OS compared to GEM/NAB-P [HR 0.79 (0.59-1.05)], PEFG [HR 0.88 (0.54-1.43)], nor the combination of GEM/erlotinib/bevicizumab [HR 0.78, (0.55-1.11)].

**Table 2:** Indirect comparisons of available treatments for advanced pancreatic cancer

**Indirect comparison**	**Overall survival**	Survival gain over OS**	Progression free survival**	**Progression free survival gain**
	**HR (95% CI)**	**Months (95% CI)**	**HR (95% CI)**	**Months (95% CI)**
***FOLFIRINOX vs.***				
Gemcitabine	0.57 (0.45-0.72)*	4.22 (2.12-6.92)*	0.59 (0.37-0.47)*	3.73 (0.98-6.48)*
Gemcitabine + NAB-P	0.79 (0.59-1.05)	1.46 (-0.27-3.81)	0.68 (0.51-0.91)*	1.54 (0.32-3.16)*
Gemcitabine + Oxaliplatin	0.66 (0.50-0.88)*	2.83 (0.76-5.59)*	0.60 (0.43-0.85)*	2.17 (0.58-4.43)*
Gemcitabine + Capecitabine	0.70 (0.53-0.92)*	2.42 (0.48-5.0)	0.58 (0.45-0.74)*	2.40 (1.14-4.01)*
Gemcitabine + Cisplatin	0.58 (0.43-0.78)*	4.06 (1.62-7.3)	0.46 (0.34-0.62)*	3.94 (2.03-6.53)*
Gemcitabine +5FU	0.70 (0.49-0.97)*	2.45 (0.17-5.63)*	0.61 (0.44-0.84)*	2.11 (0.63-4.14)*
Gemcitabine + 5FU/FA	0.55 (0.40-0.74)*	4.62 (1.93-8.28)*	n/a	n/a
Gemcitabine + pemetrexed	0.58 (0.43-0.74)*	4.03 (1.51-7.41)*	n/a	n/a
Gemcitabine + irinotecan	0.55 (0.39-0.76)*	4.62 (1.76-8.54)*	n/a	n/a
PEFG	0.88 (0.54-1.43)	0.80 (-1.66-4.76)	0.92 (0.57-1.48)	0.28 (-1.07-2.47)
Gemcitabine + exatecan	0.58 (0.43-0.79)*	4.03 (1.44-7.53)*	n/a	n/a
Gemcitabine + erlotinib	0.70 (0.51-0.94)*	3.94 (1.47-3.94)*	0.61 (0.45-0.82)*	2.11 (0.72-3.96)*
Gemcitabine + erlotinib + bevacizumab	0.78 (0.55-1.11)	1.57 (-0.55-4.61)	0.63 (0.45-0.88)*	1.95 (0.44-4.08)*
Gemcitabine + axinitib	0.56 (0.40-0.80)*	4.36 (1.40-8.54)*	0.47 (0.33-0.66)*	3.76 (1.70-6.68)*
Gemcitabine + tipifarnib	0.59 (0.44-0.79)*	3.94 (1.47-7.27)*	0.64 (0.48-0.86)*	1.82 (0.54-3.54)*
Gemcitabine + marismastat	0.58 (0.40-0.83)*	4.13 (1.18-8.38)*	0.49 (0.35-0.70)*	3.37 (1.41-6.15)*
Gemcitabine + Sorafenib	0.45 (0.28-0.73)*	6.89 (2.11-14.6)*	0.45 (0.28-0.72)*	4.00 (1.30-8.32)*
Gemcitabine + Cetuximab	0.54 (0.40-0.71)*	4.82 (2.24-8.25)*	0.44 (0.33-0.58)*	4.21 (2.41-6.56)*
Capecitabine + erlotinib	0.68 (0.46-1.00)	1.08 (0.67-6.30)	0.60 (0.88-1.30)	0.76 (0.43-2.20)

*Statistically significant, n/a = comparison not available.

Hazard ratios for overall survival and progression free survival for FOLFIRINOX with relation to all included treatments in the network-meta-analysis are shown. **Survival gain/PFS gain calculated from [(Gemcitabine median OS/HR)-Gemcitabine median OS]/[(Gemcitabine median PFS/HR)-Gemcitabine median PFS].

GEM/NAB-P was amongst the top-ranked for OS (Mean Rank 3.8/20) (Additional file 3: Figure S3). It was associated with a statistically significant benefit in survival over GEM alone (HR 0.72, 95% CI 0.62-0.82); GEM/cisplatin (HR 0.73, 95% CI 0.59-0.91); GEM/5FU/FA (HR 0.69, 95% CI 0.54-0.88); GEM/pemetrexed (HR 0.73, 95% Cl 0.58-0.93); GEM/exatecan (HR 0.73, 95% CI 0.57-0.94); GEM/cetuximab (HR 0.68, 95% CI 0.55-0.84) and GEM/sorafenib (HR 0.56, 95% CI 0.36-0.88) (Figure 3). GEM/NAB-P had a median increase of OS time of 2.2 months (95% CI 1.1-3.4) over GEM alone.PEFG had improved OS (HR 0.65, 95% CI 0.43-0.98) with median survival gain of 3 months (95% CI 0.1-7 months) over GEM alone. It was associated with statistically superior HR over GEM/cetuximab, GEM/irinotecan, GEM/5FU/FA, and GEM/sorafenib (Figure 3).

The combination of GEM/erlotinib/bevacizumab was associatedm with improved survival over GEM alone, as well as GEM/irinotecan, GEM/sorafenib, GEM/5FU/FA and GEM/cetuximab (Figure 3). It was ranked fourth for OS, with a mean rank of 4.3 (Additional file 3: Figure S3).GEM/erlotinib (without bevacizumab) was associated with improved survival over GEM alone (survival gain 1.2 months, 95% CI 0.11-2.57) and over GEM/cetuximab (Figure 3).GEM/capecitabine had statistically longer survival than GEM alone, GEM/sorafenib, GEM/5FU/FA, and GEM/cetuximab. It was associated with statistically worse OS compared to FOLFIRINOX (HR 1.43, 95% CI 1.09-1.89). No other significant differences were observed for the remaining combination chemotherapy treatments presented in Figure 3.

### Results from the network meta-analysis of the secondary outcome

The results from the indirect comparisons of FOLFIRINOX to GEM and the other included treatments, for the secondary outcome, PFS, are displayed in Table 2. FOLFIRINOX was associated with statistically significant hazard ratio for PFS over thirteen treatments, with the exception of GEM/NAB-P, GEM/erlotinib/bevacizumab, GEM/pemetrexed, GEM/irinotecan and PEFG (Table 2). FOLFIRINOX had a 63.1% probability of being best and had a mean rank of 1.38 for PFS (Additional file 4: Figure S4). GEM/NAB-P was associated with statistically significant hazard ratio for PFS in comparison to GEM alone and GEM/cisplatin.

### Sensitivity analysis of the primary outcome using network meta-analysis

In order to address possible heterogeneity between trial populations with regards to covariates such as trial sample size, year of publication, stage mix, and performance status, subgroup analyses were performed for the primary outcome, OS. Overall, results closely resembled the results presented in the primary network meta-analysis with similar effect estimates and rankings. Additional file 5: Table S1 indicates the included and excluded trials for each of the sensitivity analyses. Specifically, for the subgroup of trials including at least 100 patients per arm (n = 19 trials), FOLFIRINOX, GEM/NAB-P and GEM/erlotinib/bevacizumab were the top-ranked treatments where FOLFIRINOX was associated with statistically significant hazard ratio for OS over all treatments except for GEM/NAB-P (HR: 0.85, 95% Cl 0.47-1.33) and GEM/erlotinib/bevacizumab (HR: 0.90, 95% Cl 0.41-1.48). Similar findings were observed for the subgroup of RCTs published after 2007. In the sensitivity analysis excluding trials with a significant proportion of non-metastastic, locally advanced disease (proportion with metastases < 80%), 15 trials were included. FOLFIRINOX was associated with statistically significant hazard ratio for 9 out of 15 possible combinations, not including GEM/NAB-P, GEM/erlotinib/bevacizumab, gem/erlotinib, gem/oxaliplatin and gem/capecitabine.

Finally, the sensitivity analysis for poor performance status excluded seven trials where the proportion of patients with ECOG ≥ 0-1 (or KPS <90) was less than 15%. GEM/NAB-P was ranked first for OS and was associated with statistically significant hazard ratio for survival over GEM alone (HR 0.72, 95% CI (0.54-0.95)). GEM/erlotinib/bev and GEM/bev were also associated with significant OS over GEM alone. Both FOLFIRINOX and PEFG were excluded from this analysis. No other significant associations were observed in the remaining comparisons.

### Assessment of safety using indirect comparisons of regimens

An *a priori* decision was made to assess grade 3–4 neutropenia, febrile neutropenia, fatigue, diarrhea, sensory neuropathy and vomiting, in a Bayesian network meta-analysis. Odds ratios and 95% credible limits were obtained for each grade 3–4 toxicity comparison where treatments were ranked in order of highest toxicity rates to lowest based on the odds ratios found in the comparisons. Overall, GEM was found to be associated with the smallest risk for grade 3–4 toxicities evaluated in this study.

Additional file 6: Figure S5 summarizes the odds ratios of adverse outcomes in patients treated with either GEM/NAB-P or FOLFIRINOX, the top-ranked regimens in this analysis. Some important findings include the significantly increased odds for grade 3–4 neutropenia observed in patients treated with FOLFIRINOX compared to those treated with GEM/NAB-P (OR 1.92, 95% CL 1.10-3.39), GEM/cisplatin, GEM/capecitabine, GEM/tipifarnib and GEM/erlotinib/bevacizumab. PEFG was associated with statistically significant increased odds for neutropenia relative to the majority of the other combination treatments.

FOLFIRINOX, GEM/NAB-P, GEM/Pemetrexed and GEM/Irinotecan were associated with significantly greater odds for grade 3–4 febrile neutropenia compared to GEM alone. GEM/pemetrexed was associated with the greatest risk for grade 3–4 febrile neutropenia with statistically significant odds ratios over GEM alone, GEM/NAB-P, GEM/cisplatin, and GEM/oxaliplatin. It was not statistically different than FOLFIRINOX. No other treatments were found to be associated with statistically significant odds ratios relative to each other in this analysis.

For grade 3–4 diarrhea, GEM/oxaliplatin, GEM/cisplatin, GEM/pemetrexed, FOLFIRINOX, Gem/NAB-P and GEM/erlotinib were associated with significantly greater odds for diarrhea compared to GEM alone. In this analysis, GEM/erlotinib/bevacizumab had the lowest risk for grade 3–4 diarrhea.

GEM/NAB-P had greater odds of grade 3–4 fatigue over seven other treatments: GEM/tipifarnib, GEM/exatecan; GEM/oxaliplatin; GEM/erlotinib; GEM/capecitabine; GEM/cetuximab; GEM/erlotinib/bevacizumab. It was not statistically different than GEM/pemetrexed, PEFG, GEM/cisplatin or FOLFIRINOX, although it trended towards increased risk for grade 3-4 fatigue in comparison to FOLFIRINOX (OR: 1.90, 95% CL 0.94-3.88) (Additional file 6: Figure S5).

GEM/cisplatin, GEM/oxaliplatin, GEM/cetuximab and cisplatin/exatecan were associated with greater odds of grade 3–4 vomiting over GEM alone. GEM/cisplatin and GEM/oxaliplatin also had greater odds of vomiting relative to PEFG, GEM/marismastat, GEM/5FU and GEM/tipifarnib. GEM/exatecan and FOLFIRINOX had greater odds of vomiting over GEM/tipifarnib. Grade 3–4 vomiting in patients treated with GEM/NAB-P was not evaluable as data was not available.

FOLFIRINOX was ranked worse for grade 3–4 sensory neuropathy out of six treatments with available data (GEM/oxaliplatin, GEM/cisplatin, GEM/tipifarnib and GEM/NAB-P). All included treatments in the analysis had statistically significant increased risk for grade 3–4 sensory neuropathy compared to GEM alone. However, none of them were found to be associated with statistically significant odds ratios over other included combination therapies.

Overall, there were no statistically significant differences in odds ratios for febrile neutropenia, fatigue, diarrhea or sensory neuropathy between FOLFIRINOX versus GEM/NAB-P with the exception of grade 3–4 neutropenia (Additional file 6: Figure S5).

## Discussion

In the absence of head-to-head comparisons, we performed a network meta-analysis that evaluates the efficacy and tolerability of current treatments available for advanced pancreatic cancer, including four new combination regimens that have emerged since previously published meta-analyses [11,24,28,29]. Our study found that in comparison to GEM alone, there was statistically significant improved survival associated with FOLFIRINOX, PEFG, GEM/NAB-P, GEM/capecitabine, GEM/erlotinib with or without bevacizumab and GEM/oxaliplatin. Furthermore, in comparison to other GEM-based doublets included in this analysis, our ranking found that FOLFIRINOX, PEFG, GEM/NAB-P, GEM/erlotinib with or without bevacizumab, GEM/capecitabine and GEM/oxaliplatin were also associated with better survival. We found that, although combination therapies generally improve survival outcomes in patients with metastatic pancreatic cancer, they were also associated with greater odds for grade 3/4 adverse events over GEM alone. In particular, FOLFIRINOX and PEFG were both associated with significantly greater odds for adverse events including grade 3/4 neutropenia and grade 3/4 diarrhea. Gem/NAB-P, was found to be associated with the highest risk for grade 3/4 fatigue relative to other combination therapies included in the analysis.

Initial efforts to combine GEM with other therapies in the form of doublets have lead to a stream of statistically negative trials that were redeemed only through meta-analysis suggesting some benefit for combination with platinums or capecitabine. In contrast, recent large multi-center trials offer promising results with regimens including FOLFIRINOX [29] and GEM/NAB-P [28]. In selecting some of these regimens (FOLFIRINOX, PEFG), investigators have abandoned the traditional stepwise approach of adding a single new agent to assess the specific contribution of that agent to outcome. This approach, while obscure from a regulatory and purely scientific perspective, has met with clinically meaningful success. However, survival benefits with these more aggressive yet still palliative treatment must be weighed against the associated increased toxicities.

Although other systematic reviews and meta-analyses have been conducted to evaluate chemotherapy regimens in advanced pancreatic cancer, they have only reflected results of direct comparisons and information about safety and treatment rankings are limited and pre-date the recent phase III trials in this setting that evaluated treatments such as FOLFIRINOX, GEM/NAB-P, GEM/erlotinib/bevacizumab and erlotinib/capecitabine [3,41-50]. Therefore, it is often difficult to determine the most effective treatment. Unique to this analysis, Bayesian statistics were used to accomplish a mixed-treatment analysis where high-quality information on the effectiveness and safety of each treatment was achieved.

Results from previously published systematic reviews are similar to our own findings where meta-analyses have reported the combination of GEM with capecitabine as being significantly associated with a survival benefit compared to GEM alone [3,45] as well as the combination of GEM/erlotinib [50], which we also found in both our direct and indirect comparisons. A comparison of our own findings to those of published systematic reviews assessing the survival benefits of more recent combination treatments, including FOLFIRINOX and GEM/NAB-P is not yet possible as most published reviews pre-date the more recent phase III trials. However, a Cochrane review protocol was published in June 2013 that will compare both single agent and combination chemotherapy in pancreatic cancer [48].

A previously published meta-analyses reported that patients with ECOG performance status 0–1 had greater benefit from combination treatment while patients with worse performance did not [41]. This result may help explain why the exclusion of trials with more than 85% of patients with an ECOG performance status 0–1 resulted in more conservative treatment effects where GEM/NAB-P and GEM/erlotinib +/-bev, were associated with improved OS compared to GEM alone but no other combination treatments. Confounding may explain these findings, as patients with ECOG performance status 0–1 are often healthier, younger and more likely to tolerate more aggressive regimens such as PEFG or FOLFIRINOX. Therefore, the results in the RCTs that included a large proportion of patients with high performance status may not be representative, and further research assessing the performance of such combination treatments in a more heterogeneous population in terms of performance status is needed.

The intent of this network meta-analysis is to provide an overall impression of the benefits and risks of these chemotherapeutic options for the first-line treatment of metastatic pancreatic cancer on a relative scale, using hazard ratios as the preferred time-to-event measures. Results from this network meta-analysis may guide physicians in the recommendations of different treatments in the absence of head to head comparisons. There are, however, limitations to this approach that warrant cautious interpretation of the results. Factors such as trial heterogeneity, bias and inconsistency can affect the estimates reported in the study [51]. For instance, this analysis was performed on the assumption of consistency where the validity of indirect comparisons was determined by the extent of clinical and methodological trial similarity. However, inconsistency remains a methodological issue of multiple treatment comparisons, as it arises from pooling the data and small number of trials available for the different comparisons resulting in discrepancies between the direct and indirect comparisons, and consequently threatening the validity of the results [51-53]. In the case of our network meta-analysis, the indirect estimates were often very similar to those obtained in the direct comparisons because only single comparisons were available for the majority of the cases. This resulted in a less conventional geometry, where our network of trials did not have closed pathways (Figure 2).

Also, differences in study populations, interventions, trial design, and outcomes definitions introduce potential confounding and bias to the analysis, where baseline differences in trial populations may have affected the outcomes. For example, FOLFIRINOX included better prognosis patients (99% ECOG performance status of 0–1) and had a larger majority of males in the study, which may have altered effect estimates. In attempt to adjust for possible differences in trial populations, sensitivity analyses were performed. Results and effect estimates were comparable to results from the network meta-analysis. However, when treatments were analyzed in a sensitivity analysis for performance status, FOLFIRINOX and PEFG were excluded from the analysis, and consequently, GEM/NAB-P was identified as the optimal treatment in our rankings.

Another limitation of our analysis was that not all adverse event outcomes of interest were reported consistently across trials, particularly for febrile neutropenia and sensory neuropathy. Furthermore, there were cases where no events had occurred for the outcome of interest resulting in the requirement to add a continuity correction to the results [53]. Furthermore, not all outcomes were assessed using network meta-analysis due to missing data or different reporting methods, such as the case of quality-of-life where it could not be adequately assessed using network meta-analysis. In general, missing data resulted in wider credible intervals due to greater uncertainty around the estimates. Furthermore, dose adjustment of FOLFIRINOX is frequently required due to adverse events, such that a variety of modified FOLFIRINOX regimens are widely used in clinical practice. However, no trials exist to compare these various modified FOLFIRINOX schedules to GEM alone, creating some uncertainty about the efficacy and toxicity of the modified schedules. In light of the limitations discussed above, these results should be interpreted alongside differences in absolute effects such as survival gain in months and hazard ratios should be used as guides for physicians and not as definitive values.

## Conclusions

In conclusion, this study suggests that the use of combination therapy in the treatment of advanced pancreatic cancer may offer a greater survival benefit over GEM alone. It also allowed for the indirect comparison between combination therapies, including recent regimens, where head-to-head comparisons have not been available. GEM doublets such as GEM/capecitabine and GEM/oxaliplatin, GEM/erlotinib as well as GEM-based three or four drug regimens such as GEM/erlotinib/bevacizumab and PEFG and finally more recent treatments such as FOLFIRINOX and GEM/NAB-P all have achieved statistically significant survival benefits over GEM alone as well as several other combination therapies.

Whether these treatments should be tested in a large multi-center randomized clinical trial, or whether the choice of treatment is left to the physician’s discretion, is the subject of debate. To fully elucidate the comparative effectiveness, rigorously conducted comparative studies among more similar populations, or network meta-regression analyses of patient-level data are required. Further, given the differences in costs of treatments, a cost-effectiveness analysis is warranted. Nonetheless, the application of network meta-analysis in this setting can help inform current therapeutic decision-making and direct the design of future studies.

## Abbreviations

5FU: Fluorouacil; FOLFIRINOX: Folinic acid plus 5-fluorouacil plus Irinotecan plus oxaliplatin; GEM: Gemcitabine; HR: Hazard ratio; NAB-P: 130 nm albumin-bound paclitaxel; NMA: Network meta-analysis; ORR: Overall Response Rate; OS: Overall Survival; PEFG: Gemcitabine + epirubicin + cisplatin + 5FU; PFS: Progression Free Survival; RCT: Randomized Controlled Trial; Sup.: Supplemental.

## Competing interests

The authors declare that they have no competing interests.

## Authors’ contributions

GG and DJ designed the review. GG conducted the review assisted by GA, DJ and SG. CC provided statistical guidance for the network meta-analysis. GG wrote the manuscript with comments on drafts from DJ, CC, SG and GA. All authors read and approved the final manuscript.

## Pre-publication history

The pre-publication history for this paper can be accessed here:

http://www.biomedcentral.com/1471-2407/14/471/prepub

## Supplementary Material

Additional file 1: Figure S1 Forest plot of pairwise comparisons for overall survival of individual trials. Click here for file

Additional file 2: Figure S2 Forest plot of hazard ratios for overall and survival (left) and progression free survival (right) for all comparisons of FOLFIRINOX with other treatments included in network meta-analysis. Click here for file

Additional file 3: Figure S3 Mean rank for overall survival for treatments included in Bayesian network meta-analysis. *A low mean rank indicates the greatest overall survival relative to other treatments. Gem = gemcitabine; 5FU = 5-flurouacil; PEFG = gemcitabine + epirubicin + 5FU + cisplatin; NAB-P = NAB-Paclitaxel; bev = bevacizumab; erlo = erlotinib; cape = capecitabine; oxali = oxaliplatin; tipif = tipifarnib; pem = pemetrexed; cis = cisplatin; irino = irinotecan; FA = folinic acid; cetux = cetuximab; soraf = sorafenib*. Click here for file

Additional file 4: Figure S4 Probability of a treatment being best out of 100% for overall survival and progression free survival. Click here for file

Additional file 5: Table S1 Summary of included/excluded studies in a priori sensitivity analyses. Click here for file

Additional file 6: Figure S5 Forest plot of adverse outcomes in patients treated with either gemcitabine + NAB-P or FOLFIRINOX where an odds ratio >1 indicates higher risk of toxicities for patients treated with FOLFIRINOX. Click here for file
