# Supplementary figures and images for: Chemotherapy regimens for advanced pancreatic cancer: a systematic review and network meta-analysis
Source: BMC Cancer. 2014 Jun 27;14:471. doi: 10.1186/1471-2407-14-471 (PMC4097092; doi:10.1186/1471-2407-14-471)

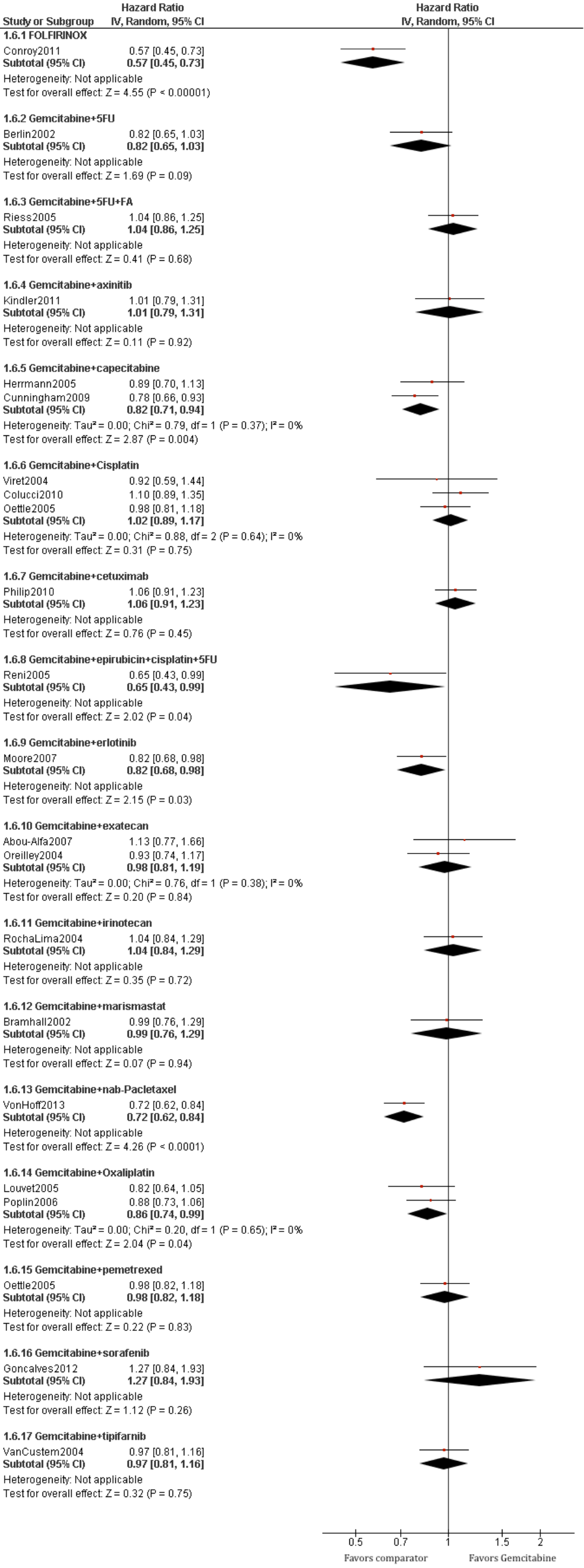

Supplement: Additional file 1: Figure S1 — Forest plot of pairwise comparisons for overall survival of individual trials. [file 1471-2407-14-471-S1.pdf]

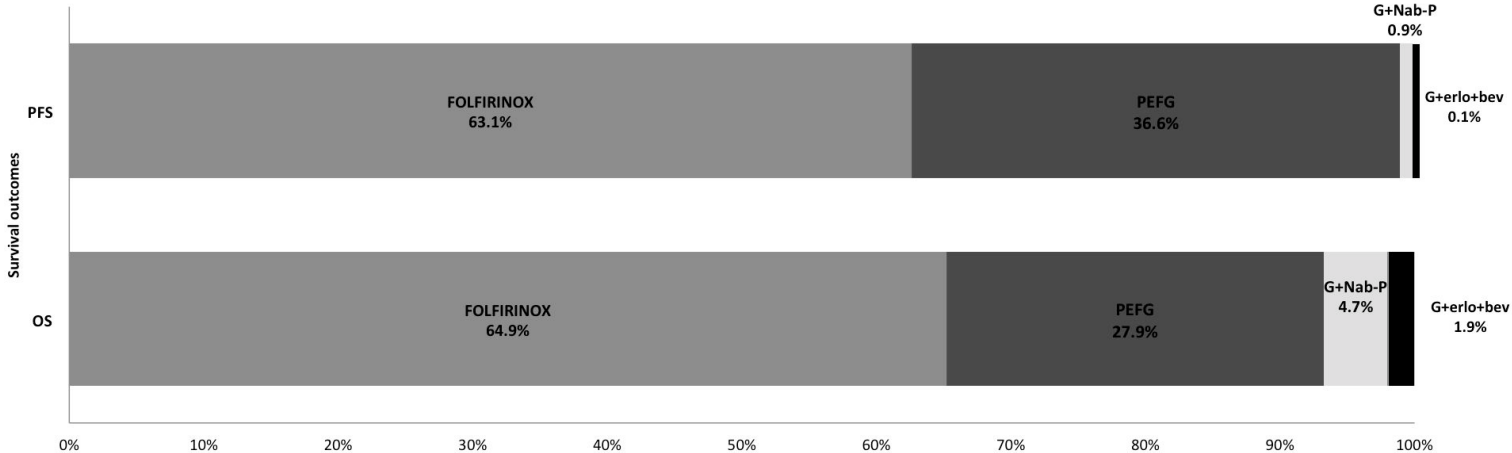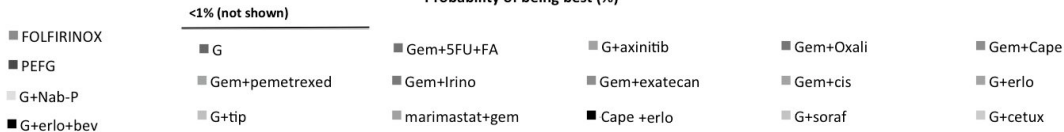

Supplement: Additional file 2: Figure S2 — Forest plot of hazard ratios for overall and survival (left) and progression free survival (right) for all comparisons of FOLFIRINOX with other treatments included in network meta-analysis. [file 1471-2407-14-471-S2.pdf]

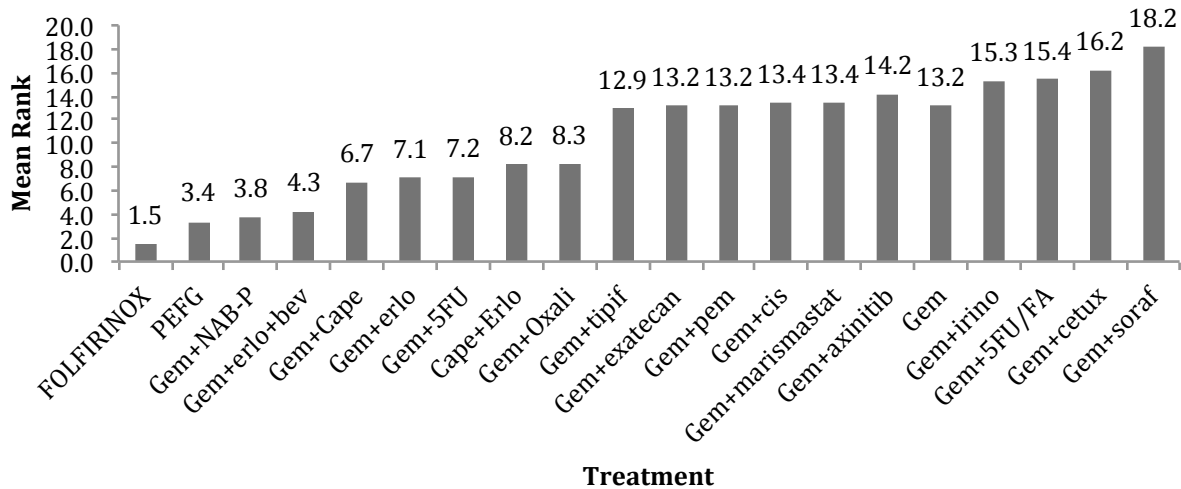

Supplement: Additional file 3: Figure S3 — Mean rank for overall survival for treatments included in Bayesian network meta-analysis. A low mean rank indicates the greatest overall survival relative to other treatments. Gem = gemcitabine; 5FU = 5-flurouacil; PEFG = gemcitabine + epirubicin + 5FU + cisplatin; NAB-P = NAB-Paclitaxel; bev = bevacizumab; erlo = erlotinib; cape = capecitabine; oxali = oxaliplatin; tipif = tipifarnib; pem = pemetrexed; cis = cisplatin; irino = irinotecan; FA = folinic acid; cetux = cetuximab; soraf = sorafenib. [file 1471-2407-14-471-S3.pdf]

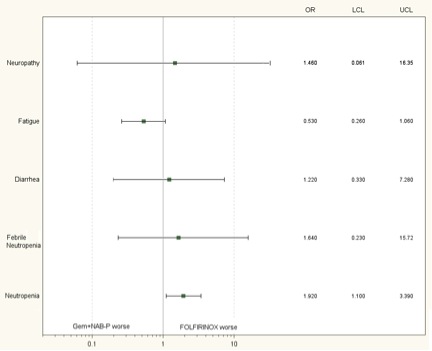

Supplement: Additional file 5: Table S1 — Summary of included/excluded studies in a priori sensitivity analyses. [file 1471-2407-14-471-S5.jpeg]
