# Supplementary material for: Chemotherapy regimens for advanced pancreatic cancer: a systematic review and network meta-analysis
Source: BMC Cancer. 2014 Jun 27;14:471. doi: 10.1186/1471-2407-14-471 (PMC4097092; doi:10.1186/1471-2407-14-471)
Supplement: Additional file 6: Figure S5 — Forest plot of adverse outcomes in patients treated with either gemcitabine + NAB-P or FOLFIRINOX where an odds ratio >1 indicates higher risk of toxicities for patients treated with FOLFIRINOX. [file 1471-2407-14-471-S6.pdf]

Additional file 6: Table S1. Summary of included/excluded studies in a priori sensitivity analyses.

Author (year)	Experimental Treatment	**Included in sensitivity analysis (Y/N):**			
		Year of publication >2007	Number of patients/arm >100	Proportion Stage IV >75%	Proportion ECOG 0-1** <85%
Bramhall (2002)	Gemcitabine +Marismastat	N	Y	N	N
Berlin (2002)	Gemcitabine+5FU	N	Y	N	Y
VanCustem (2004)	Gemcitabine +Tipifarnib	N	Y	Y	Y
Rocha Lima (2004)	Gemcitabine+ Irinotecan	N	Y	Y	Y
Louvet (2005)	Gemcitabine +Exatecan	N	N	N	Y
Reni (2005)	Gemcitabine + Oxaliplatin	N	N	N	N
Riess (2005)	Gemcitabine +epirubicin +cisplatin+5FU	N	Y	Y	Y
Oettle (2005)	Gem+ Capecitabine	N	Y	Y	N
Abou-Alfa (2006)	Gem+Pemetrexed	N	Y	Y	Y
Heinemann (2006)	Gemcitabine +Cisplatin	N	N	Y	N
Stathopoulos (2006)	Gemcitabine +Irinotecan	N	N	N	N
Poplin (2006)	Gemcitabine + Oxaliplatin	N	Y	Y	Y
Herrmann (2007)	Gemcitabine+5FU	N	Y	Y	Y

	+folinic acid				
Moore (2007)	Gemcitabine +Erlotinib	N	Y	Y	Y
Cunningham (2009)	Gem + Capecitabine	Y	Y	N	Y
VanCustem (2009)	Gem+Erlotinib+ Bevacizumab*	Y	Y	Y	Y
Philip (2010)	Gemcitabine + Cetuximab	Y	Y	Y	Y
Colucci (2010)	Gem+Cisplatin	Y	Y	Y	Y
Kindler (2011)	Gem+Axinitib	Y	Y	N	N
Conroy (2011)	FOLFIRINOX	Y	Y	Y	N
Goncalves (2012)	Gem+Sorafenib	Y	N	N	N
Heinemann (2012)	Capecitabine+ Erlotinib*	Y	Y	Y	Y
Von Hoff (2013)	Gem+ NAB-P	Y	Y	Y	Y

All experimental treatments in table were compared to gemcitabine alone. **Comparator gemcitabine+erlotinib.****If KPS was reported en lieu of ECOG performance status, the equivalent value (KPS >80%) was used instead.*
